# Open-loop analysis on sympathetically mediated arterial pressure and urine output responses in rats: effect of renal denervation

**DOI:** 10.1186/s12576-020-00759-w

**Published:** 2020-06-25

**Authors:** Toru Kawada, Yohsuke Hayama, Takuya Nishikawa, Satoru Suehara, Satoshi Sawada, Tetsuo Tanaka, Minako Uenohara, Masaru Sugimachi

**Affiliations:** 1 Department of Cardiovascular Dynamics, National Cerebral and Cardiovascular Center, Osaka, 564-8565 Japan; 2 Corporate R&D Center, Terumo Corporation, Kanagawa, 259-0151 Japan

**Keywords:** Pressure diuresis, Sympathetic nerve activity, Arterial pressure, Open-loop analysis, Equilibrium diagram

## Abstract

Primary acute sympathetic activation (PASA) can increase arterial pressure (AP). Under this situation, the kidneys may receive mutually opposing influences from sympathetic activation: a direct anti-diuretic effect via the renal innervation and pressure diuresis. We examined whether PASA would reduce urine output regardless of the AP elevation. We also examined the impact of renal denervation (RDN) on urine output during PASA. The experiment was performed on rats 3 to 9 days after unilateral RDN (*n* = 10). Under anesthesia, systemic sympathetic nerve activity (SNA) was varied over a wide range via the carotid sinus baroreflex. The slope of urine flow versus SNA was positive (0.252 ± 0.052 μL·min^−1^·kg^−1^· %^−1^) on the intact side, and it was greater on the denervated side (0.331 ± 0.069 μL·min^−1^·kg^−1^· %^−1^, P < 0.05). In conclusion, urine output change was an effect of elevated AP during PASA. Nevertheless, RDN was able to augment pressure diuresis during PASA.

## Background

**Fig. 1:**
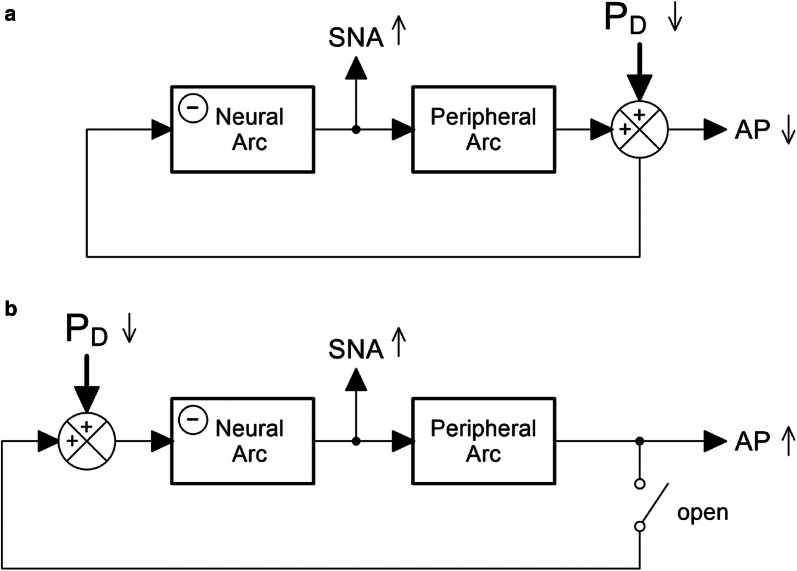
Schema of the arterial baroreflex system. The baroreflex system may be divided into neural arc and peripheral arc subsystems. The negative sign in the neural arc indicates signal inversion through the neural arc. Under a baroreflex closed-loop condition (**a**), a decrease in arterial pressure (AP) due to an exogenous perturbation (P_D_) induces reflex activation of sympathetic nerve activity (SNA), which counteracts the effect of P_D_. Under this condition, the AP reduction and the reflex sympathetic activation act synergistically to reduce urine output. However, changes in AP and SNA are not always reciprocal. When SNA increases via a central command, an AP elevation ensues. We refer to this latter situation as primary acute sympathetic activation (PASA). This situation may be mimicked under a baroreflex open-loop condition by imposing P_D_ on the isolated baroreceptor regions (**b**). During PASA, the kidneys may receive mutually opposing influences from the increased SNA (an anti-diuretic effect through renal innervation) and the increased AP (a diuretic effect through an increase in renal perfusion pressure). If urine output decreases during PASA, the urine output change can be interpreted as a cause for the AP elevation. Conversely, if urine output increases during PASA, the urine output change needs to be interpreted as an effect of the AP elevation

The arterial baroreflex system is an essential negative feedback system that stabilizes arterial pressure (AP) against pressure fluctuations. This system may be analyzed by dividing it into two principal subsystems [1]: the neural arc subsystem that describes the relationship between a baroreceptor pressure input and efferent sympathetic nerve activity (SNA), and the peripheral arc subsystem that describes the relationship between SNA and AP. When AP is decreased by an exogenous perturbation, SNA increases via the negative feedback through the neural arc and counteracts the pressure decrease (Fig. 1a). In this situation, the AP reduction and the reflex sympathetic activation act synergistically to reduce urine output because the AP reduction decreases the renal perfusion pressure and the sympathetic activation exerts a direct anti-diuretic effect via the renal innervation [2]. However, changes in AP and SNA may not always be reciprocal. As an example, when SNA increases via a central command, an AP elevation ensues. We refer to this latter situation as primary acute sympathetic activation (PASA) in this paper to indicate that sympathetic activation occurs first, followed by the secondary AP elevation. During PASA, the kidneys may receive mutually opposing influences from sympathetic activation: the direct anti-diuretic effect via the renal innervation and the pressure diuresis resulting from the AP elevation.

The urine output control during PASA does not seem to be well understood. If urine output decreases during PASA, the urine output change can be interpreted as a cause for the AP elevation. Conversely, if urine output increases during PASA, the urine output change needs to be interpreted as an effect of the AP elevation. To analyze changes in urine output during PASA, we employed a baroreflex open-loop procedure in anesthetized rats [3]. Under the baroreflex open-loop condition, pressure fluctuations applied to the isolated baroreceptor regions induces the same directional changes in SNA and AP (Fig. 1b), which allowed the analysis of the urine output change during PASA. The experiment was performed on rats 3 to 9 days after unilateral renal denervation (RDN), and urine output was compared between the intact and denervated sides.

## Methods

### Ethical approval

Male Wistar–Kyoto (WKY) rats were purchased from Japan SLC. The rats were cared for in strict accordance with the Guiding Principles for the Care and Use of Animals in the Field of Physiological Sciences, which has been approved by the Physiological Society of Japan. The Animal Subjects Committee at the National Cerebral and Cardiovascular Center reviewed and approved all experimental protocols.

### Renal denervation

Unilateral RDN was performed on 14 rats (372 ± 28 g, mean ± SD) using a sterile preparation under isoflurane anesthesia. Through a flank incision, visible renal nerves were sectioned under a dissecting microscope, and a solution of 10% phenol in ethanol was painted around the renal vessels [4, 5]. After the surgery, butorphanol tartrate was injected intramuscularly for post-operative analgesia. Each rat was housed individually and given free access to standard laboratory chow and water.

### Acute experiment

3 to 9 days after RDN, the rats were anesthetized by intraperitoneal injection (2 mL/kg) of a mixture of urethane (250 mg/mL) and α-chloralose (40 mg/mL), and mechanically ventilated with oxygen-enriched room air. The anesthetic mixture was diluted 18-fold with physiological saline and infused via the right femoral vein (2 mL·kg^−1^·h^−1^). Ringer lactate solution was infused continuously (4 mL·kg^−1^·h^−1^) via the left femoral vein for fluid maintenance. Systemic AP was measured from the right femoral artery. The body temperature of the animal was maintained at approximately 38 °C by using a heating pad and a lamp.

A postganglionic branch of the left splanchnic sympathetic nerve was exposed for SNA recording. A pair of stainless-steel wire electrodes (AS633, Cooner Wire, CA, USA) were attached to the nerve and fixed with silicone glue (Kwik-Sil, World Precision Instruments, FL, USA). The SNA signal was full-wave rectified and low-pass filtered at a 30-Hz cut-off frequency. The noise level was determined after intravenous injection of a ganglionic blocker hexamethonium bromide (60 mg/kg) at the end of the experiment. The splanchnic sympathetic nerve was selected as a proxy of systemic SNA because the control of splanchnic vascular resistance is essential to systemic AP regulation [6]. Further, our previous study indicated that the static input–output relationship of the neural arc did not differ significantly between the splanchnic and renal SNAs in normal rats [7]. The laterality of SNA was not examined in the present study.

**Fig. 2:**
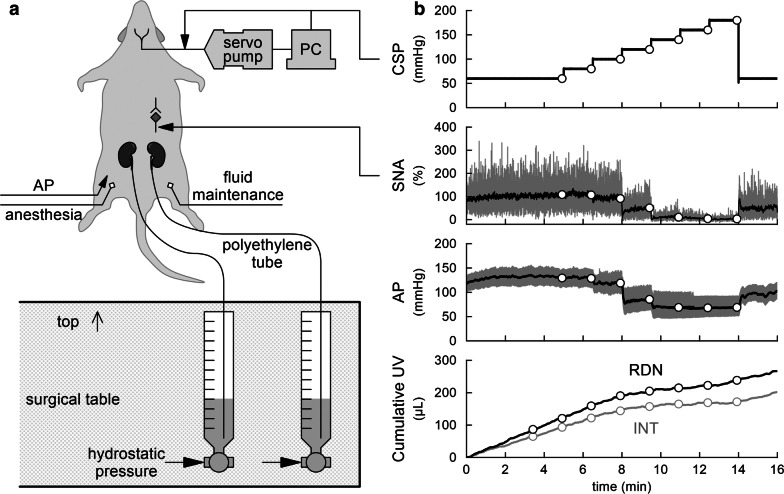
**a** Schema of experimental settings. Carotid sinus pressure (CSP) was controlled via a servo-pump system. Urine output was assessed from the hydrostatic pressure of cumulated urine. PC, personal computer. **b** An example time series obtained from one rat. CSP was changed in a stepwise manner with a step duration of 90 s. Sympathetic nerve activity (SNA) and arterial pressure (AP) decreased in response to the CSP elevations. Gray and black lines in the SNA plot indicate 10-Hz resampled and 2-s moving average signals, respectively. Gray and black lines in the AP plot indicate 200-Hz resampled and 2-s moving average signals, respectively. Urine volume (UV) is displayed as a 10-Hz resampled signal. The UV was greater on the renal denervation (RDN) side than the intact (INT) side. The open circles indicate 10-s averaged values used for data analysis

Each ureter was cannulated with a polyethylene tube (KN-392-SP 8, inner diameter: 0.2 mm, outer diameter: 0.5 mm, Natsume, Japan) via a horizontal abdominal incision. Urine was collected in a 1-mL syringe placed vertically on the lateral side of a surgical table with its top just below the surface of the table (Fig. 2a). The hydrostatic pressure of the collected urine was measured and calibrated against that of 1-mL physiological saline.

Bilateral carotid sinus baroreceptor regions were isolated from the systemic circulation [8, 9]. Reflexes other than the carotid sinus baroreflex were minimized by sectioning the aortic depressor nerves and vagal nerves. The carotid sinus pressure (CSP) was changed from 60 to 180 mmHg in increments of 20 mmHg [3] using a servo-controlled piston-pump system (ET-126, Labworks, Costa Mesa, CA) connected to catheters inserted into the common carotid arteries. The CSP step duration was either 60 or 90 s. The step duration was longer than the total response time of the urine flow (UF) change in response to a short-term AP perturbation in rats (ranged from 17.6 to 26.7 s) [10]. The negative feedback loop of the carotid sinus baroreflex was opened, and the function of the carotid sinus baroreflex was tested over a wide input pressure range. We call this analysis as “open-loop analysis” of the carotid sinus baroreflex. The negative feedback loop of the cardiopulmonary reflex was also opened, in a sense, by sectioning the vagal nerves, but we did not test the function of the system. The cardiopulmonary reflex was simply disabled.

Atrial stretch is another modality to test diuretic and natriuretic responses without significantly changing AP [11]. Left but not right atrial stretch significantly affects urine output, which suggests that the effects of left atrial stretch may be mediated via neural or humoral mechanisms other than atrial natriuretic peptide release [12]. Since we aimed to clarify the interaction between the effect of AP change and the neural control of the kidneys, we used the carotid sinus baroreflex to vary SNA and AP over a wide range.

After the induction of anesthesia, it took approximately 15 min for venous and arterial catheterizations (fluid maintenance was started), approximately 15 min for the SNA recording procedure, approximately 30 min for the bilateral carotid sinus baroreceptor isolation procedure, and approximately 30 min for bilateral cannulations to the ureters. After the completion of the surgical procedure, another 30 min was allowed before starting the CSP step input protocol.

### Blood, urine, and renal tissue samples

At the end of the baroreflex experiment, blood and cumulated urine from each kidney were sampled and frozen at − 80 °C. The sodium and creatinine concentrations of the blood and urine samples were measured by outsourcing to Hachioji laboratories (SRL Inc., Japan). The rats were euthanized with an overdose of intravenous sodium pentobarbital. The kidneys were removed, and a section of each kidney (150–200 mg) was frozen at − 80 °C. Later, thawed renal tissue was homogenized in Tris buffer (pH 8.6), and the norepinephrine concentration was measured using a liquid chromatography system (Eicom, Japan). The norepinephrine measurements were necessary because the RDN procedure was incomplete in some rats, probably due to the failure of dissecting the renal nerves running through tissues between the renal artery and vein. Successful RDN was defined arbitrarily as a reduction of more than 90% in tissue norepinephrine concentration in the denervated side compared to the intact (non-denervated) side. Among 14 rats, ten met this criterion, and we hereafter report the data from these ten rats. Consequently, renal tissue norepinephrine concentrations were 80.0 ± 11.2 and 2.3 ± 0.7 ng/g in the intact and denervated sides, respectively (mean ± SE, *n* = 10 rats). Seven rats received right RDN, and three rats left RDN. If the observed difference in urine output was attributable solely to the laterality, including the data from denervation on the different side would cancel the statistical difference.

### Data analysis

Data were digitized at 1000 Hz using a 16-bit analog-to-digital converter. A preliminary analysis indicated that the number of postoperative days did not significantly correlate with the slope of UF versus AP. The difference in the CSP step duration (60 or 90 s) did not significantly affect the slope of UF versus AP, either. Accordingly, data from all ten rats were analyzed as a single cohort.

In each rat, the SNA and AP values at each CSP were obtained as averages during the last 10 s of each step. The SNA was normalized with the value at CSP of 60 mmHg (100%) and the value after the ganglionic blockade (0%). The UF (in μL/min) at each step was derived from an increment of the urine volume (in μL) from the preceding step. When the step duration was 90 s, the increment was divided by 1.5. The normalized urine flow (nUF, in μL·min^−1^·kg^−1^) was then obtained as UF divided by the body weight of the rat.

The characteristics of the baroreflex total reflex arc (AP versus CSP) and neural arc (SNA versus CSP) were described by the four-parameter logistic function [13]:

1 $$y = \frac{{P_{1} }}{{1 + exp\left[ {P_{2} \left( {CSP - P_{3} } \right)} \right]}} + P_{4},$$

where *P*_1_ is the response range, *P*_2_ is the slope coefficient, *P*_3_ is the midpoint pressure on the CSP axis, and *P*_4_ is the minimum value of the sigmoid curve.

The characteristics of the baroreflex peripheral arc (AP versus SNA) were quantified by linear regression. The operating-point SNA and AP were determined from the intersection between the fitted neural and peripheral arcs on a baroreflex equilibrium diagram [3, 14, 15].

The relationships of nUF versus SNA and nUF versus AP were analyzed by linear regression. The absolute values of the intercept and slope may be different from those under conscious conditions because of anesthesia [4]. The nUF at the operating-point AP was estimated using the regression line of nUF versus AP. Creatine clearance was calculated from the nUF at the operating-point AP and creatinine concentrations in the plasma and cumulated urine. Fractional sodium excretion was calculated from the creatinine and sodium concentrations in the plasma and cumulated urine. Statistical comparisons between the intact and denervated sides were performed using Wilcoxon signed-rank test [16].

## Results

Stepwise increases in CSP progressively decreased SNA and AP (Fig. 2b). The urine volume increased as time elapsed. On each side, the change in urine volume from 0 to 8 min (when the mean AP was above 100 mmHg) was steeper than that from 9 to 14 min (when the mean AP was below 100 mmHg). At each time point, the urine volume was greater on the denervated than the intact side.

**Fig. 3:**
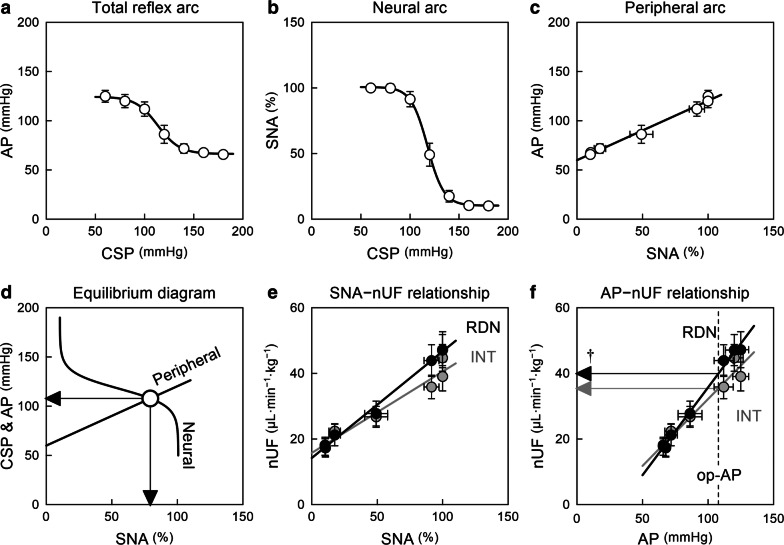
Group-averaged static characteristics of the total reflex arc (**a**), neural arc (**b**), and peripheral arc (**c**) of the carotid sinus baroreflex. *CSP* carotid sinus pressure, *AP* arterial pressure; *SNA* sympathetic nerve activity. **d** The baroreflex equilibrium diagram constructed from the fitted neural and peripheral arcs. Downward and leftward arrowheads indicate the operating-point AP and SNA, respectively. **e** The relationship of normalized urine flow (nUF) versus SNA in the intact (INT) and renal denervation (RDN) sides. **f** The relationship of nUF versus AP in the INT and RDN sides. The vertical dashed line indicates the operating-point AP (op-AP). The horizontal arrowheads indicate nUF at the operating-point AP. ^†^*P* < 0.01 by Wilcoxon signed-rank test. Data are expressed as mean ± SE values (*n* = 10 rats)

Figure 3 and Table 1 summarize the open-loop static characteristics of the carotid sinus baroreflex. The total reflex arc (Fig. 3a) and the neural arc (Fig. 3b) approximated inverse sigmoid curves. The peripheral arc (Fig. 3c) approximated a straight line. In the baroreflex equilibrium diagram (Fig. 3d), the intersection between the neural and peripheral arcs provides the operating point.

**Table 1:** Parameters of the carotid sinus baroreflex open-loop characteristics

Total reflex arc	
*P*_1_, response range, mmHg	57.8 ± 6.6
*P*_2_, slope coefficient, mmHg^−1^	0.142 ± 0.021
*P*_3_, midpoint pressure, mmHg	113.3 ± 4.1
*P*_4_, minimum pressure, mmHg	65.0 ± 1.7
Neural arc	
*P*_1_, response range, %	91.6 ± 3.1
*P*_2_, slope coefficient, mmHg^−1^	0.142 ± 0.015
*P*_3_, midpoint pressure, mmHg	117.8 ± 3.6
*P*_4_, minimum value, %	9.5 ± 2.6
Peripheral arc	
Intercept, mmHg	59.0 ± 2.4
Slope, mmHg/ %	0.609 ± 0.052
Operating-point parameters	
Operating-point SNA, %	80.8 ± 3.8
Operating-point AP, mmHg	107.4 ± 3.6

Data are expressed as mean ± SE (*n* = 10 rats)

*SNA* sympathetic nerve activity, *AP* arterial pressure

There was a positive correlation between SNA and nUF on both sides (Fig. 3e), and the regression slope was significantly greater on the denervated side (Table 2). Likewise, nUF positively correlated with AP on both sides (Fig. 3f), and the regression slope was significantly greater on the denervated side. The vertical dotted line in Fig. 3f indicates the operating-point AP determined from the equilibrium diagram. The nUF at the operating-point AP, shown by the leftward arrowhead, was significantly higher on the denervated than the intact side (Table 2).

**Table 2:** Effects of unilateral renal denervation (RDN) on normalized urine flow (nUF)

	INT	RDN
Slope of nUF versus SNA, μL·min^−1^·kg^−1^· %^−1^	0.252 ± 0.052	0.331 ± 0.069^*^
Intercept of nUF versus SNA, μL·min^−1^·kg^−1^	15.6 ± 2.8	13.9 ± 4.0
Slope of nUF versus AP, μL·min^−1^·kg^−1^·mmHg^−1^	0.420 ± 0.081	0.552 ± 0.112^†^
Intercept of nUF versus AP, μL·min^−1^·kg^−1^	−7.4 ± 5.7	−16.5 ± 8.1
nUF at the operating-point AP, μL·min^−1^·kg^−1^	36.1 ± 3.5	41.0 ± 4.1^†^

Data are expressed as mean ± SE (*n* = 10 rats)

*INT* intact side, *SNA* sympathetic nerve activity, *AP* arterial pressure

^*^P < 0.05 and ^†^*P* < 0.01 by Wilcoxon signed-rank test

Sodium and creatinine concentrations in the plasma and cumulated urine were measured in nine out of ten rats (Table 3). Creatinine clearance, calculated using nUF at the operating-point AP, did not differ significantly between the intact and denervated sides (2662 ± 352 vs. 2908 ± 472 μL·min^−1^·kg^−1^, *n* = 9). Fractional sodium excretion did not differ significantly between the intact and denervated sides (0.55 ± 0.13% vs. 0.61 ± 0.14%, *n* = 9).

**Table 3:** Sodium and creatinine concentrations in plasma and urine

	Plasma	INT urine	RDN urine
Sodium, mEq/L	142.4 ± 0.7	47.0 ± 6.2	52.1 ± 7.9
Creatinine, mg/dL	0.39 ± 0.02	29.8 ± 3.6	29.1 ± 3.7

Data are expressed as mean ± SE (*n* = 9 rats). There was no significant difference in the sodium or creatinine concentration between the intact and denervated sides by Wilcoxon signed-rank test

*INT* intact side, *RDN* renal denervation side

## Discussion

### Sympathetic activation and pressure diuresis

Sympathetic activation exerts an anti-diuretic effect via renin release, renal vasoconstriction, and sodium and water reabsorption [2]. Beers et al. [17] reported that UF negatively correlated with graded increases in renal SNA under a constant renal perfusion pressure, which demonstrates a direct anti-diuretic effect of SNA. However, the renal perfusion pressure can change with AP during PASA. In the present study, nUF increased as SNA increased (Fig. 3e), which suggests that the urine output change was not a cause for the AP elevation during PASA. The pressure diuresis overrode the direct anti-diuretic effect of SNA during PASA (Fig. 3f). Renal vascular resistance and stressed blood volume might be increased to elevate AP, but the urine output reduction did not contribute to the AP elevation during PASA. The observation is different from the situation of long-term sympathetic activation, which is associated with fluid retention [18]. Time course of renin release, subsequent generation of local angiotensin II, and promotion of proximal tubular sodium reabsorption may be possible factors explaining the difference between the acute and chronic effects of sympathetic activation.

In conscious rats, Steele et al. [19] reported that the pressure–diuresis relationship usually exhibited a positive slope (16/24 observations), but occasionally also exhibited a slope not significantly different from zero (6/24 observations) and even a negative slope (2/24 observations). The fact that a negative pressure–diuresis relationship was infrequent suggests that pressure diuresis can usually override the direct anti-diuretic effect of SNA during acute fluctuations of AP and UF.

### Effects of RDN on the relationship of nUF versus AP

We did not actively control the hydration status of the rat. Fluid maintenance, started from the beginning of the surgical preparation and lasted for nearly 2 h before the initiation of the CSP step input protocol, might have reduced the inter-individual difference in hydration status to a certain extent. Nevertheless, the regression slope of nUF versus AP showed a large variance among the rats (Table 2), which may reflect the difference in hydration status. Plasma osmolarity was not measured, but the plasma sodium concentration was within a normal range at the end of the baroreflex study (Table 3). RDN did not significantly affect renal function, as assessed by creatinine clearance and fractional sodium excretion, in the present experimental settings.

The effect of RDN on urine output was not uniform, but depended on the SNA level (Fig. 3e). As predicted, RDN did not significantly affect nUF when SNA was suppressed beforehand by baroreflex activation. On the other hand, RDN significantly increased nUF when SNA was increased by baroreflex unloading. Urine output under baroreflex closed-loop conditions may be determined from nUF at the operating-point AP (Fig. 3f). The increase in nUF at the operating-point AP on the denervated side is in agreement with the results of a study by Rogenes and Gottschalk [4] in which renal function was examined in rats with unilateral RDN.

The effect of RDN on long-term AP remains unanswered in the present study. According to a study by Jacob et al. [20], the AP of Sprague–Dawley rats was significantly lower in a bilateral RDN group than a sham-operated group. In that study, water intake and urine output did not differ between RDN and sham-operated groups under a normal salt diet, which indicates that water intake and urine output can be maintained despite a lower operating-point AP after bilateral RDN. However, conflicting results have also been reported. Kline et al. [21] reported that bilateral RDN did not affect systolic AP for 7 weeks post-surgery compared to sham operation in WKY rats. Herlitz et al. [5] reported significantly higher urine volume in WKY rats during a 10-day post-RDN observation period compared to sham-operated controls. Further research is required to delineate the relationship between acute and chronic effects of RDN on pressure diuresis and resulting water balance and AP regulation.

## Limitations

The vagal nerves were sectioned to establish open-loop conditions for the carotid sinus baroreflex. Vagal afferents play an important role in volume loading-induced low-pressure baroreflex activation [22]. Hepatic receptors also contribute to body fluid homeostasis [23]. Further studies are clearly required for an integrated understanding of AP regulation during PASA via the diverse mechanisms of fluid homeostasis.

## Conclusions

We demonstrated that urine output increases with increasing SNA and AP during PASA. The result indicates that the urine output change was not a cause but an effect of the AP elevation during PASA. Nevertheless, RDN was able to augment the pressure diuresis during PASA.

## Data Availability

The datasets used and/or analyzed during the current study are available from the corresponding author on reasonable request.

## Abbreviations

AP: Arterial pressure
CSP: Carotid sinus pressure
nUF: Normalized urine flow
PASA: Primary acute sympathetic activation
RDN: Renal denervation
SNA: Sympathetic nerve activity
UF: Urine flow
WKY: Wistar–Kyoto
